# Clinical nurses’ work-life balance prediction due to patient safety incidents using classification and regression tree analysis: a secondary data analysis

**DOI:** 10.1186/s12912-024-01719-0

**Published:** 2024-01-25

**Authors:** Jiwon Kang, Soon-Sun Kwon, Youngjin Lee

**Affiliations:** 1 Department of Family, Health and Wellbeing, University of Minnesota Extension, 1420 Eckles Ave, St Paul, MN 55108 US; 2 College of Nursing, Ajou University, 164 World Cup-Ro, Yeongtong-Gu, Suwon, 16499 South Korea; 3 Departments of Mathematics and Department of Artificial Intelligence, College of Natural Sciences, Ajou University, 164 World Cup-Ro, Yeongtong-Gu, Suwon, 16499 South Korea

**Keywords:** Patient safety, Safety management, Work-life balance, Occupational stress

## Abstract

**Background:**

Patient safety incidents lead to performance difficulties for nurses when providing nursing practice. This affects work-life balance and causes second and third-victimization. This study predicts factors affecting clinical nurses’ work-life balance due to patient safety incidents using classification and regression tree analysis techniques.

**Methods:**

This study was a secondary analysis of data from a cohort research project, which used a descriptive survey for data collection. Participants comprised 372 nurses. Data were collected using SurveyMonkey, a mobile-based survey software solution, from January to September 2021. Data included the general characteristics of clinical nurses, second damage, second damage support, third damage, and work-life balance. The specific variables included in the analysis chosen through rigorous Lasso analysis form the foundation for predicting work-life balance. Variables with low explanatory power were excluded, thereafter, the variables selected by Lasso were analyzed with a classification and regression tree model to predict work-life balance.

**Results:**

A regression tree was applied to predict work-life balance using seven variables—education level, marital status, position, physical distress, second-victim support, turnover intentions, and absenteeism (selected through Lasso analysis). After pruning, at tree size four, when turnover intentions were < 4.250, physical distress < 2.875, and second-victim support < 2.345, the predicted work-life balance was 3.972. However, when turnover intentions were < 4.250, physical distress < 2.875, and second-victim support ≥ 2.345, then the predicted work-life balance was 2.760.

**Conclusions:**

This study's insights offer crucial groundwork for crafting targeted workforce risk management strategies and fostering a conducive organizational culture to mitigate nursing occupational stress, potentially curbing the recurrence of patient safety incidents and improving nursing practice while enhancing patient outcomes. Future research should explore second and third victim experiences across various healthcare settings globally to understand their impact on WLB and patient safety outcomes.

## Background

When healthcare workers provide medical care to patients, physical and mental injuries or side effects can occur [1] and cause patient safety incidents (PSIs), where patients unintentionally become the first victims in unavoidable circumstances [2]. Furthermore, the nurses become second-victims as they are traumatized by the unexpected PSIs, which negatively impacts their daily lives. These cascading effects cause harm and losses in healthcare facilities, resulting in third-victim experiences [3].

Experiencing patients’ pain and deaths resulting from PSI causes personal, emotional, and professional difficulties for nurses who provide care, thereby leading to additional problems, such as second-victimization [4–7]. Negative emotions, such as fatigue, anxiety, depression, and low self-confidence caused by patient safety events directly affect organizations’ productivity losses through turnover intentions and absenteeism, which could continue as a vicious cycle that threatens patient safety [8]. Furthermore, the continuous workload of nurses, without any improvements in their working environments, such as manpower and salaries, and the resultant shortage of nurses increases third-victims’ experiences, such as turnover intentions and absenteeism [5, 9–11].

Work-life balance (WLB) is a state of harmony between work, family, and social activities outside work according to one’s life priorities [12]. Failure to maintain WLB not only causes deterioration of physical and mental health, but also decreases the quality of life of individuals and their families, reduces job satisfaction and organizational commitment, and increases employee turnover [13]. As the degree of second and third victims’ experiences is an important variable in predicting clinical nurses’ WLB, it is necessary to understand its relationship with each variable. Exploring the relationship between nurses' Patient Safety Incidents (PSIs), second and third-victim experiences, and achieving Work-Life Balance (WLB) is crucial. Understanding how PSIs impact nurses emotionally, professionally, and personally is crucial, as it significantly influences patient care quality, organizational dynamics, and the well-being of healthcare workers. Addressing this link presents an opportunity to introduce focused support strategies, potentially decreasing turnover rates, enhancing job satisfaction, and cultivating a safer patient care environment within healthcare organizations [14]. Understanding how nurses are emotionally, professionally, and personally impacted provides an opportunity to bolster their support and enhance the organizational environment. These efforts ultimately contribute to improving patient safety, treatment outcomes, and the overall quality of healthcare services [15].

To improve the quality of care and maintain a culture of patient safety, second-victims should be supported, and research and support strategies should be initiated and evaluated as the patient safety movement progresses [7]. A strategy to prevent third damage by reducing second damage will also be an effective strategy for not only improving organizational medical service quality and productivity but also for improving job satisfaction, engagement, and patient safety [16]. Additionally, supporting one’s WLB—one of the driving forces supporting joy in the job process—should be prioritized as a strategy to achieve workforce development and health goals [10]. Therefore, the link between second-victims’ experiences and support, and third-victims’ experiences need to be examined to establish strategies for improving nurses’ performance and appropriately managing patient safety issues.

Most previous studies on nurses’ second-victim experiences from PSIs examined the causality of the impact of nurses’ perceived second-victim experiences on third-victim experiences [3, 17]. Although these studies tried to establish the relationship between variables through causality, their research results had limitations, which explains the complex patterns inherent in the relationship between second- and third-victims, and WLB for healthcare workers, especially nurses. Therefore, it is necessary to develop a model that can accurately evaluate the impact of factors such as PSI second-victims’ experiences, second-victims’ support, and third-victims’ experiences on nurses’ WLB, and closely examine the influence of these variables.

The target variables in this study were selected to predict WLB using the Least absolute shrinkage and selection operator (Lasso) regression model and the results were derived using the algorithm of classification and regression tree (CART) analysis. The lasso model is to handle collinearity and variable selection to find an accurate model among the predictor variables. The CART analysis is a valuable tool to provide an insightful understanding of complex and hierarchical relations and guide nurses to reduce gaps in the application of evidence to practice [18]. In order to find an easy-to-interpret and accurate model for the relationship between WLB and predictor variables in data with collinearity and missingness, Lasso and CART analyses are applied.

This study aimed to 1) analyze the relationship between second and third-victim experiences caused by PSIs, and the WLB of clinical nurses were predicted using classification and regression tree analysis techniques; and 2) present effective supportive measures to reduce second- and third-victimization, improve nurses’ work conditions, and ultimately enhance their WLB by identifying the factors involved in this relationship.

## Methods

### Design

This study was a secondary analysis of data from a cohort research project (AJIRB-SBR-SUR-20–328), which aimed to longitudinally investigate the impact of compassion competence on the job outcomes and patient safety outcomes among clinical nurses. This study comprised a retrospective data analysis to explore the PSI-related second-victim experiences, support for second-victims, and third-victim experiences among clinical nurses and their WLB, as well as examine their interrelationships. This study was approved by Ajou University Hospital’s Institutional Review Board (IRB) (approval id: AJIRB-MED-SUR-21–585) and conducted in accordance with the Code of Ethics of the World Medical Association (Declaration of Helsinki). The participants who provided written consent confirmed their understanding of the study’s purpose and method. Further, they were assured that they could refuse to participate at any time during the study without any disadvantage and that the collected data would be kept anonymous and used only for research purposes.

### Participants

This study’s participants comprised clinical nurses, who were recruited via poster advertisements on employee bulletin boards, homepage banners for research, social media, and word of mouth. The inclusion criteria of nurses were (a) presently working in acute general hospitals, with a capacity of over 100 beds in South Korea, (b) providing direct care for patients, and (c) having over three months of nursing service experience. Nurses primarily involved in administrative tasks were excluded. Of the 443 questionnaires investigated between January and September 2021, 406 were completed (a response rate of 91.6%). Thereafter, 34 questionnaires were excluded since the respondents had no PSI experience and those who had, missed answering some of the critical variables. Thus, data from 372 nurses were included in the final analyses, to implement follow-up evaluations in the second year of the longitudinal compassion cohort study.

### Measurements

#### Second-victim Experience and Support Tool (SVEST)

The Second-victim Experience and Support Tool (SVEST) developed by Burlison et al. [5] to assess second-victims’ experiences, second-victims’ support systems, and negative work-related PSI outcomes among healthcare providers were used after obtaining permission from the developer and Korean validation research’s author [19], who adapted the tool. In this study, 29 items of the original tool were equally measured. Based on the previous study [19], the seven dimensions’ scores of the 29 items were finally divided into three subdomain total scores (second-victim experience, second-victim support, and third-victim experience). The tool includes reverse-coded items, and uses a 5-point Likert scale, from 1 “strongly disagree” to 5 “strongly agree.” A higher score indicates greater second-victim and third-victim experiences.

##### Second-victim experiences

Second-victim experiences were represented by the mean scores for psychological distress, physical distress, and reduced professional self-efficacy (each of which comprised four items). Higher scores indicate greater mental distress, physical distress, and professional self-efficacy. The reliability of the tool as measured with Cronbach’s α was 0.79–0.87.

##### Second-victim support

Second-victim support was represented by the mean scores of four items for colleague support, four items for supervisor support, three items for institutional support, and two items for non-work-related support. Higher scores indicate higher levels of support from colleagues, supervisors, institution, and non-work-related friends. The reliability of the tool as measured with Cronbach’s α was 0.61–0.87.

##### Third-victim experiences

Third-victim experiences were represented by the mean scores for turnover intentions and absenteeism, both comprising two items each. Higher scores indicate higher turnover intentions and absenteeism. The reliability of the tool as measured with Cronbach’s α was 0.81–0.88.

#### Work-life balance scale (WLB)

The Work-Life Balance Scale developed by Kim and Park [20] was used. It consists of 29 items in four domains: eight items for work-family balance, eight items for work-leisure balance, nine items for work-growth balance, and four items for work-general balance. Each item is rated on a 5-point Likert scale, from 1 “Strongly agree” to 5 “Strongly disagree.” Although in the original study, higher scores indicate poorer WLB, we reverse-coded each item during statistical analyses for a clearer interpretation, thus, a higher total score indicated better WLB. The tool’s Cronbach’s α was 0.68–0.85 and 0.74, respectively.

### Data analysis

In the WLB data set, WLB and many explanatory variables exist, and there is a correlation between these variables. We aim to find an accurate and interpretable prediction model from the data with collinearity and missingness applying Lasso and CART models. Lasso and CART models can achieve the purpose of the study in that they solve collinearity between predictor variables based on a regression model and find an interpretable prediction model. To predict WLB with explanatory variables, the analysis proceeded with the subsequent steps. As the first step, to minimize the error of the regression model, Lasso analysis was performed, and variables with low explanatory power were excluded. In the second step, variables in the final model selected through Lasso were analyzed with a regression tree to predict WLB, the target variable. The description of each analysis method is as follows:

Lasso is a regression analysis method that involves both variable selection and regularization to enhance the prediction accuracy and interpretability of the resulting statistical model [21]. This is well-suited for models showing high levels of multicollinearity or variable selection (or parameter elimination). The Lasso model’s performance was assessed using Root Mean Squared Prediction Error (RMSPE), which estimates the mean residual, and Mean Absolute Prediction Error (MAPE), which refers to the unexplained standard error of predictions obtained by using the model [22].

CART (a variant of the decision tree) is a predictive model for classification and prediction that aims to predict continuous outcomes from the predictor variables. It refers to a regression model method that determines the relationship between one dependent variable and a series of independent variables that split off from the initial data set. It also indicates that the target variable is present, and an algorithm is used to predict its value.

All statistical analyses were performed using glmnet and tree packages in the R (version 4.2 & 4.1.1) (R Foundation for Statistical Computing, Vienna, Austria. ISBN 3–900,051-07–0, URL http://www.r-project.org) using the stats package [23–25]. All statistical tests were two-tailed, confidence intervals (CI) were considered significant when they did not include zero, and *p*-values < 0.05 were considered significant.

### Validity, reliability, and rigor

Nurses' perceived second-victim experiences were measured using four items each from the SVEST for Psychological distress, Physical distress, and Professional self-efficacy. The Cronbach’s α of the tool was 0.79–0.87 at the time of development and 0.73 (0.43–0.90) in this study.

Nurses' perceived second-victim support was measured using four items for colleague support, four items for supervisor support, three items for institutional support, and two items for non-work-related support from the SVEST. The tool’s Cronbach’s α was 0.61–0.87 at the time of development and 0.56 (0.28–0.77) in this study.

Nurses’ perceived third-victim experience was measured using two items each for turnover intentions and absenteeism from the SVEST, regarding negative work-related outcomes. The tool’s Cronbach’s α was 0.81–0.88 at the time of development and 0.70 (0.45–0.83) in this study.

## Results

### Participants’ general characteristics, second-victim experiences, third-victim experiences, and WLB

The general characteristics of the 372 participants were as follows (Table 1): Most were female and had a bachelor’s degree. Most were staff nurses, whose mean nursing experience was 7.28 ± 2.4 years, with 77.7% having more than five years of nursing experience.

**Table 1:** Relationship between general characteristics and second-victim experience, second-victim support, third-victim experience, and work-life balance (*n* = 372)

Variables	Categories	n(%)	Second-victim experience			Second-victim support			Third-victim experience			Work-life balance		
			**Mean ± SD**	**t/F**	***P (scheffe)***	**Mean ± SD**	**t/F**	***p***	**Mean ± SD**	**t/F**	***p***	**Mean ± SD**	**t/F**	***p***
Gender	Male	14(3.8)	2.90 ± .93	-2.091	.037	3.13 ± .53	-.296	.767	2.38 ± 1.02	-1.585	.114	2.88 ± .26	-.053	.958
	Female	358(96.2)	3.34 ± .75			3.17 ± .53			2.72 ± .79			2.90 ± .70		
Age	< 30	113(30.4)	3.30 ± .75	-.304	.761	3.20 ± .52	.811	.418	2.62 ± .76	-1.446	.149	2.93 ± .73	.497	.619
	30 ≤	259(69.6)	3.33 ± .77			3.15 ± .53			2.75 ± .82			2.89 ± .70		
Marital status	Married	153(41.1)	3.31 ± .72	-.338	.736	3.21 ± .53	1.252	.211	2.70 ± .83	-.180	.857	2.97 ± .72	1.573	.117
	Not married	219(58.9)	3.33 ± .80			3.14 ± .53			2.71 ± .79			2.85 ± .70		
Religion	Yes	148(39.8)	3.26 ± .78	-1.261	.208	3.12 ± .54	-1.268	.206	2.68 ± .81	-.505	.614	2.93 ± .70	.684	.495
	No	224(60.2)	3.36 ± .75			3.20 ± .52			2.73 ± .80			2.88 ± 7.16		
Education level	Associate^a^	88(23.7)	3.19 ± .80	4.333	.014 a,b < c	3.19 ± .46	.110	.896	2.52 ± .70	4.525	.011 a < c	2.92 ± .64	1.908	.150
	Bachelor’s ^b^	269(72.3)	3.34 ± .73			3.16 ± .54			2.75 ± .80			2.92 ± .72		
	Master/ doctoral ^c^	15(4.0)	3.80 ± .99			3.14 ± .63			3.10 ± 1.16			2.55 ± .75		
Position	Charge nurse	32(8.6)	3.26 ± .89	-.517	.605	3.47 ± .53	3.436	.001	2.58 ± .93	-.958	.339	2.77 ± .83	-1.095	.274
	Staff nurse	340(91.4)	3.33 ± .75			3.14 ± .52			2.72 ± .79			2.91 ± .70		
Nursing experience	3 years or less	16(4.3)	2.84 ± .93	3.686	.026	3.23 ± .51	.458	.633	2.61 ± .98	.170	.844	2.83 ± .78	.270	.764
	More than 3 years – less than 5 years	67(18.0)	3.41 ± .70			3.21 ± .49			2.69 ± .74			2.86 ± .72		
	More than 5 years	289(77.7)	3.33 ± .76			3.15 ± .54			2.72 ± .81			2.92 ± .70		
Work unit	Medical unit	107(28.8)	3.38 ± .66	.408	.665	3.14 ± .46	.232	.793	2.72 ± .69	2.383	.094	2.92 ± .74	.309	734
	Surgical unit	109(29.3)	3.32 ± .81			3.18 ± .59			2.58 ± 84			2.93 ± .66		
	Others	156(41.9)	3.29 ± 80			3.18 ± .52			2.79 ± .84			2.87 ± .71		
Region of hospital	Seoul metropolitan area	281(75.5)	3.33 ± .79	.406	.685	3.17 ± .54	.403	.687	2.68 ± .81	-.1358	.175	2.90 ± .70	.027	.978
	Others	91(24.5)	3.30 ± .68			3.15 ± .47			2.81 ± .79			2.90 ± .72		

There were significant differences in second-victim experiences according to gender (t = -2.091, *p* = 0.037), education levels (F = 4.333, *p* = 0.014), and nursing experience (t = 3.686, *p* = 0.026). As a result of the post-hoc analysis of education levels, the second-victim experience scores were significantly higher in participants with master’s/doctoral degrees than in those with associate and bachelor’s degrees. There were significant differences in second-victim support according to positions (t = 3.436, *p* < 0.01). Third-victim experience significantly differed according to education levels (F = 4.525, *p* = 0.011), and the post-hoc analysis results revealed that the percentage of master’s/doctoral degree holders was significantly higher than associate degree holders.

### Multi-factor prediction and selection

As a first step, we used Lasso regression analysis to consider various models as explanatory variables affecting WLB. The final model that minimizes the error of regression was derived by excluding variables with low explanatory powers. We also considered the problem of multicollinearity between explanatory variables. Table 2 shows that the variables affecting WLB were selected. If the sign of the regression coefficient is positive, it implies a bad effect on WLB, and vice versa.

**Table 2:** Result of lasso regression model

Outcome	Predictors	Coefficient of predictors
**Work-life balance**	**Intercept**	3.707
	Education level	0.010
	Marital status	-0.127
	Position	-0.259
	Physical distress	0.111
	Second-victim support	-0.176
	Turnover intentions	0.185
	Absenteeism	-0.081
R^2^ = 0.194, RMSPE = 0.277, MAPE = 0.182		

### Predictions of WLB

As a second step, we applied the regression tree to predict WLB with seven variables selected through Lasso analysis. The preferred strategy was to grow a large tree and stop the splitting process only on reaching some minimum node size (usually four or five) [26]. The *prune tree* module gives a graph on the number of nodes versus deviance, based on cost complexity pruning.

**Fig. 1:**
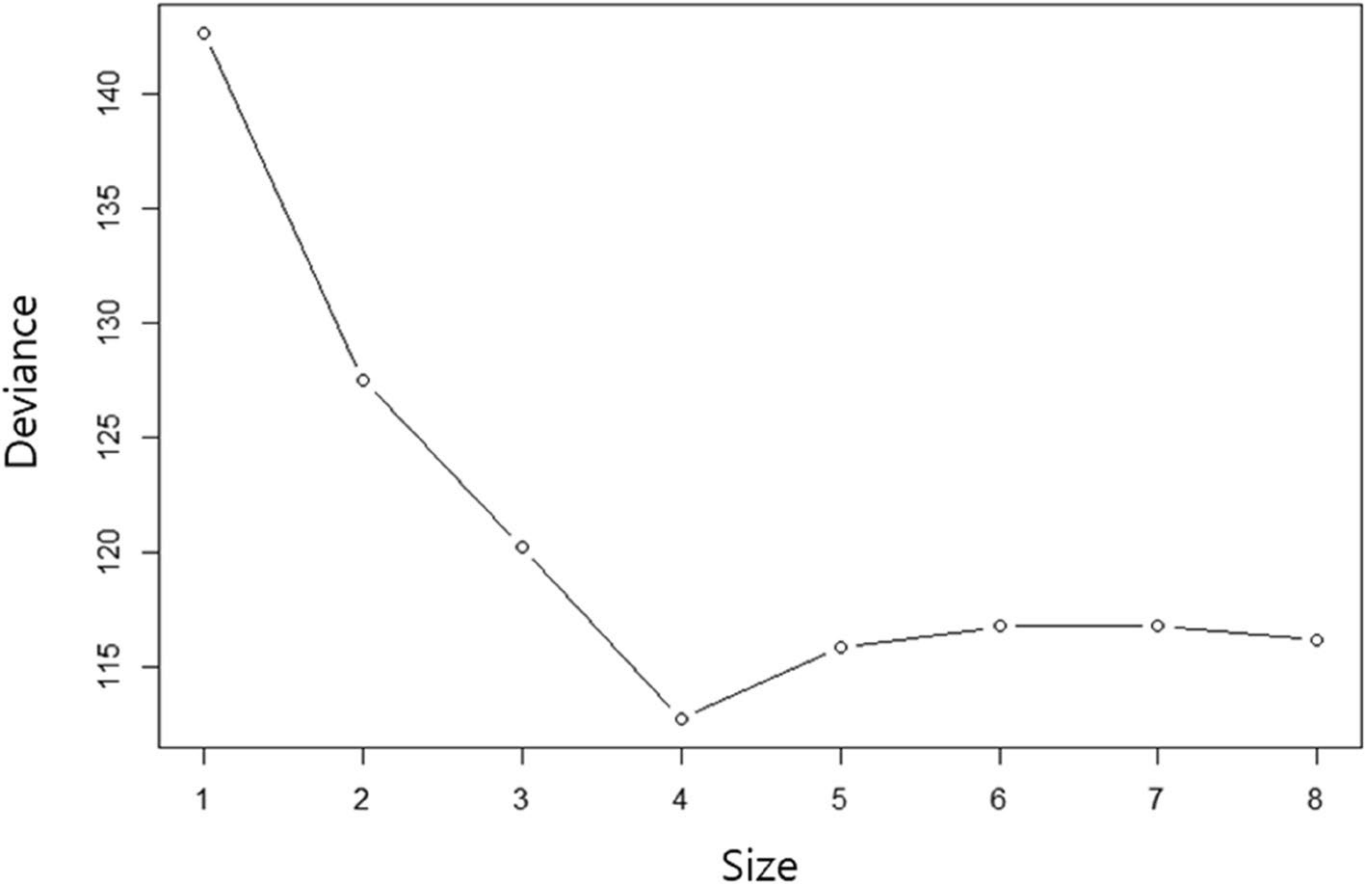
Scree plot of optimal tree size

**Fig. 2:**
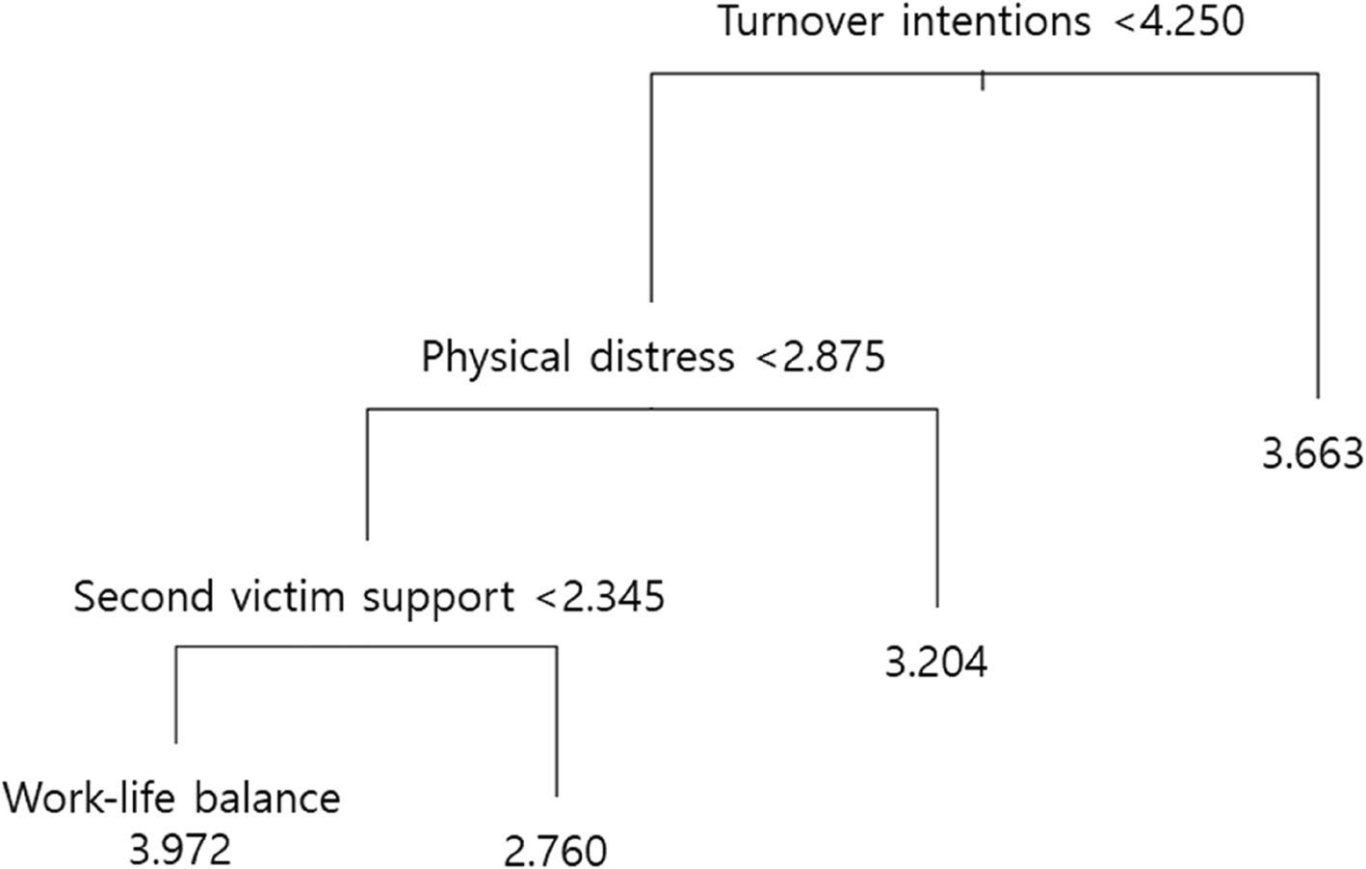
Result of the regression tree

Figures 1 and 2 show the scree plot and results of the regression tree at tree size four, respectively, and the decision tree was found to be an upside-down schema, which means the root was at the top and then this root was split into several nodes. In other words, the upside-down approach refers to the process of starting from the top with the whole data and gradually splitting the data into smaller subsets. The regression tree takes a sample of variables available (or takes all available variables at once) for splitting. A split is determined based on criteria like the Gini Index or Entropy concerning variables. If turnover is < 4.250, physical distress < 2.875, and second-victim support < 2.345, then the WLB is high, and the predicted WLB is 3.972. However, if turnover intentions are < 4.250, physical distress is < 2.875 and second-victim support is ≥ 2.345, then the predicted WLB is 2.760. The power of models based on Lasso and CART analysis could be explained as RMSE 0.672. It could be interpreted as having an error of about 0.672 in the prediction compared to the actual value.

## Discussion

Studies have confirmed that various factors can influence WLB in nurses [12, 13]. In this study, the factors affecting WLB of nurses’ second-victim experiences, second-victim support, and third-victim experiences were analyzed in-depth, and a predictive model was identified. The degree of experiences of second- and third-victims after PSI was an important variable in predicting clinical nurses’ WLB, and among them, seven variables—education level, physical distress, turnover intentions, marital status, position, second-victim support, and absenteeism—were considered as the main explanatory variables. From among these, in the final model, the main variables affecting the effective WLB of clinical nurses were turnover intentions, physical distress, and second-victim support. Based on this study's findings, we were able to identify the key variables influencing work-life balance aligned with the research objectives. Consequently, we could delineate the pathways of factors negatively impacting work-life balance and propose suitable strategies considering risk factors.

Seven explanatory variables affecting WLB were derived based on the Lasso regression. Particularly, education levels, physical distress, and turnover intentions were found to have a negative effect on WLB. This suggests that the higher the education level, the more it could support confidence and responsibility in performing nursing tasks, based on one’s knowledge and experience, but getting frustrated in the situation of the second-victim was more likely. Moreover, experiencing an adverse event related to patient safety is a strong stressor, thus, resulting in job stress among clinical nurses [27]. These stresses include physical changes, such as tachycardia, sleep disturbance, and loss of appetite [28], that inevitably affect nurses’ health. As turnover intentions due to burnout and stress eventually lead to a shortage of manpower owing to nurse turnover, which affects patient outcomes [28]; it is important to first manage the stress and job performance of individual nurses [29] that facilitate managing second damage and avoiding progression to third damage.

This study’s results revealed that marital status, position, second-victim support, and absenteeism had a positive effect on WLB. Faced with heavy workloads and shift work, married nurses with children were constantly attempting work-family balance in the hope that they would be able to take good care of their families and raise their children [30]. This suggests that family life can affect married nurses’ WLB and absenteeism rates, as compared to unmarried nurses, who may not have family responsibilities. Therefore, it is necessary to consider granting leave effectively to improve work efficiency, reduce absenteeism, strengthen the competitiveness of hospitals, and enhance the image of organizations. Depending on the nurses’ job positions, there are differences in work that affect their individual lives. Newly qualified RNs are at increased risk of absenteeism because of illnesses related to work commitments and psychological demands. To prevent this, nursing managers must monitor nurses’ mental and physical functioning [31]. Additionally, strategies for reducing absenteeism and retaining newly graduated RN teams require input and organizational commitments from nursing managers [32]. In particular, nurses with second-victim experience were significantly more resilient than nurses who did not use peer support and support programs [33]. Peer support helps to reduce the emotional burden and provides an understanding of the error situation, thus enabling accessing the post-error event [34]. For clinical nurses who were involved in PSIs, support from colleagues, supervisors, family, and organizations reduces various difficulties caused by second-victim experiences and helps them to return to their normal daily lives and work [17].

In the decision tree, this study’s results showed the predicted WLB (target value) of four types based on three predictor variables (turnover intentions, physical distress, and second-victim support). The high value of WLB was 3.972 in the group where turnover intentions were less than 4.250, physical distress was less than 2.875, and second victim support was less than 2.345. Therefore, it can be concluded that turnover intentions, physical distress, and second-victim support are key factors influencing WLB, in that order.

Nurses’ WLB is important for nurse retention, and building organizational stability is essential for increasing job satisfaction and lowering turnover intentions [35]. Nurses’ turnover intentions have been presented as a recurring problem that can be reduced by improving their working conditions [4]. As physical afflictions such as insomnia, appetite problems, and tension-related pains are more severe in rigid work situations, a flexible time policy may solve this problem [36], but the method has not yet been fully established. It is expected that accepting such a system will take time owing to the temperament of the nurses who work in shifts. However, research related to providing an attractive work schedule and work environment is continuously being undertaken in an attempt to provide a solution [37].

While hospital-led support and mentorship opportunities are important for clinical nurses to recover from their second-victim experiences, it is also important to understand and support nurses’ needs, motivate them to become determined to recover from their wounds, and give them opportunities to develop further [29]. Organizations can implement several strategies to enhance work-life balance, nurse retention, and organizational stability. Introducing support programs for work-life balance, managing nursing workloads effectively, providing psychological support, offering appropriate rewards and incentives, enhancing learning and professionalism, and improving organizational culture and leadership can collectively contribute to achieving these objectives. These strategies aim to foster a conducive environment that promotes well-being, reduces turnover, and ensures stability within healthcare settings. Previous studies on nurses’ perceived second-victim experiences have investigated the status of these experiences, and discussions are emerging to devise strategies and programs [38, 39]. Therefore, it is necessary to improve the existing procedures and work environment by reviewing the work environment and work performance management of second-victims.

Accurate assessment of WLB based on second- and third-victims’ experiences using various methods to assess PSIs may provide better evidence for managing risk factors and remedial outcomes. Objective methods, such as health care providers’ electronic monitoring for patient safety, and modifying and developing safety protocols, are ideal for reducing second- and third-victim experiences resulting from PSI but may be costly or have no evidence of effectiveness on outcomes. Representing a predictive model by deriving variables in this way, based on the data obtained from the self-report questionnaire raises concerns about reporting bias, but since it is applicable in any environment and cost-effective, it can be an efficient way to provide additional information about one’s concerns.

### Limitations

Although we implemented cross-validation to include various predictors for WLB and check the accuracy of the tree model, since the factor classification methods of Lasso and regression trees are exploratory for data analysis, they may provide incomplete models which limit the interpretation of the results. Future studies should attempt to replicate these preliminary findings in other groups to increase the reliability and accuracy of analyses on WLB for second- and third-victims. Additionally, since the study’s participants were recruited from among nurses who worked in a general hospital in the capital area, its findings have limited generalizability to the entire nurse population. Further, the collected data were self-reported, which might be vulnerable to a response bias. However, efforts were made to reduce this bias by employing validated questionnaires and rigorous collection methods. Finally, comparing the relationship between each sub-area with the results of previous studies may be a limitation, owing to the lack of studies on the second and third damage experiences related to patient safety incidents of nurses.

The practical implication of the findings focuses on strategies to suppress the tertiary victimization effect in hospitals by positively reinforcing secondary victimization experiences. For human resource management in healthcare settings, decision-makers must contribute to reducing patient safety incidents by emphasizing the importance of organizational culture, support mechanisms, and awareness programs. The strategy established based on the results of this study can provide practical guidance for implementation in clinical practice, aligned with the broader goal of improving patient safety and health professional well-being.

## Conclusion

In conclusion, this study provided a forward-thinking perspective on the interconnectedness of individual well-being and organizational resilience in healthcare. The effects of relevant factors in WLB are based on second- and third-victim experiences following a PSI and identified the optimal factor pathways for WLB using Lasso regression and regression tree methods. PSIs not only bring about difficulties for patients and their families but also cause physical and psychological difficulties for the clinical nurses involved. Further, healthcare facilities become the third victim because of negative work-related outcomes. Clinical nurses who have more second-victim experiences were found to have more third-victim experiences and work-life imbalances.

Managing individual nurses’ stress and job outcomes is important to prevent PSIs. The findings of this study can be used to develop interventions that strengthen support from colleagues and organizations to reduce clinical nurses’ second-victim experiences, thus preventing third-victim outcomes for hospitals. By advocating for strategic interventions aimed at the secondary connector experience, this research contributes to the ongoing discourse on improving healthcare outcomes and fostering resilient healthcare institutions. The proposed recommendations have practical implications for healthcare practitioners, administrators, and policymakers seeking to fortify the fabric of healthcare delivery systems.

## Data Availability

The data sets used and analyzed during this study can be obtained from the corresponding author upon reasonable request.

## Abbreviations

PSIs: Patient safety incidents
WLB: Work-life balance
CART: Classification and regression tree
SVEST: Second-victim Experience and Support Tool
RMSPE: Root mean squared prediction error
MAPE: Mean absolute prediction error
CI: Confidence intervals

## References

[1] Patient Safety Network. Agency for Healthcare Research and Quality Improvement. 2020. https://psnet.ahrq.gov/. Accessed 15 Mar 2022.

[2] Hegarty J, Flaherty SJ, Saab MM, Goodwin J, Walshe N, Wills T (2021). An international perspective on definitions and terminology used to describe serious reportable patient safety incidents: A systematic review. J Patient Saf.

[3] Mira JJ, Lorenzo S, Carrillo I, Ferrús L, Pérez-Pérez P, Iglesias F (2015). Interventions in health organisations to reduce the impact of adverse events in second and third victims. BMC Health Serv Res.

[4] Boamah SA, Laschinger H (2016). The influence of areas of worklife fit and work-life interference on burnout and turnover intentions among new graduate nurses. J Nurs Manag.

[5] Burlison JD, Scott SD, Browne EK, Thompson SG, Hoffman JM (2017). The second victim experience and support tool: Validation of an organizational resource for assessing second victim effects and the quality of support resources. J Patient Saf.

[6] Mok WQ, Chin GF, Yap SF, Wang W (2020). A cross-sectional survey on nurses’ second victim experience and quality of support resources in Singapore. J Nurs Manag.

[7] Seys D, Scott S, Wu A, Van Gerven E, Vleugels A, Euwema M (2013). Supporting involved health care professionals (second victims) following an adverse health event: A literature review. Int J Nurs Stud.

[8] Seys D, Wu AW, Van Gerven EV, Vleugels A, Euwema M, Panella M (2013). Health care professionals as second victims after adverse events: A systematic review. Eval Health Prof.

[9] Carlesi KC, Padilha KG, Toffoletto MC, Henriquez-Roldán C, Juan MA (2017). Patient safety incidents and nursing workload. Rev Lat Am Enferm.

[10] Kelly LA, Lefton C, Fischer SA (2019). Nurse leader burnout, satisfaction, and work-life balance. J Nurs Adm.

[11] Kwon MJ (2019). The effect of turnover nurses’ social support, emotional labor and subjective health on resilience. J Ind Converg.

[12] Twenge JM, Campbell SM, Hoffman BJ, Lance CE (2010). Generational differences in work values: Leisure and extrinsic values increasing, social and intrinsic values decreasing. J Manag.

[13] Lee YS, Jang SJ (2013). The effects of work-family conflicts, organizational culture, and supervisor support, on the mental and physical health of married nurses. Health Soc Care Rev.

[14] Rehder KJ, Adair KC, Hadley A, McKittrick K, Frankel A, Leonard M, Sexton JB (2020). Associations between a new disruptive behaviors scale and teamwork, patient safety, work-life balance, burnout, and depression. Jt Comm J Qual Patient Saf.

[15] Tran Y, Liao HH, Yeh EH, Ellis LA, Clay-Williams R, Braithwaite J (2021). Examining the pathways by which work–life balance influences safety culture among healthcare workers in Taiwan: path analysis of data from a cross-sectional survey on patient safety culture among hospital staff. BMJ Open.

[16] Burlison JD, Quillivan RR, Scott SD, Johnson S, Hoffman JM (2021). The effects of the second victim phenomenon on work-related outcomes: Connecting self-reported caregiver distress to turnover intentions and absenteeism. J Patient Saf.

[17] Kim EM, Kim SA, Kim JI, Lee JR, Na SGJ (2017). Effects of nurse’s second victim experiences on third victim experiences: Multiple mediation effects of second victim supports. Qual Improv Health Care.

[18] Kuhn L, Page K, Ward J, Worrall-Carter L (2014). The process and utility of classification and regression tree methodology in nursing research. J Adv Nurs.

[19] Jung SJ, Lee Y, Bae SH (2022). Influence of clinical nurses' second-victim experience and second-victim support in relation to patient safety incidents on their work-related outcomes. J Korean Acad Nurs Adm.

[20] Kim CW, Park CYJ (2008). A study on the development of a “Work-life balance” scale. Leis Stud.

[21] Tibshirani R (1996). Regression shrinkage and selection via the lasso. J R Stat Soc B.

[22] Barnston AG (1992). Correspondence among the correlation, RMSE, and Heidke forecast verification measures; refinement of the Heidke score. Weather Forecasting.

[23] Breiman L, Friedman JH, Olshen RA, Stone CJ. Classification and regression trees. Routledge; 2017.

[24] Friedman J, Hastie T, Tibshirani R (2009). Glmnet: Lasso and elastic-net regularized generalized linear models. R package version.

[25] Hastie TJ, Chambers JM, Hastie TJ (1992). Generalized additive models. Statistical Models in S.

[26] Gareth J, Daniela W, Trevor H, Robert T. An introduction to statistical learning: With applications in R. Springer; 2017.

[27] Kakemam E, Kalhor R, Khakdel Z, Khezri A, West S, Visentin D (2019). Occupational stress and cognitive failure of nurses and associations with self-reported adverse events: A national cross-sectional survey. J Adv Nurs.

[28] Chang HY, Friesner D, Chu TL, Huang TL, Liao YN, Teng CI (2018). The impact of burnout on self-efficacy, outcome expectations, career interest and nurse turnover. J Adv Nurs.

[29] Boamah SA, Read EA, Spence Laschinger HK (2017). Factors influencing new graduate nurse burnout development, job satisfaction and patient care quality: A time-lagged study. J Adv Nurs.

[30] Kim HN, Kim JS (2016). Work-family compatibility experience of married nurse: Focusing on the expanding stage of the family life cycle. J Korea Acad Ind Coop Soc.

[31] Roelen C, van Rhenen W, Schaufeli W, van der Klink J, Magerøy N, Moen B (2014). Mental and physical health-related functioning mediates between psychological job demands and sickness absence among nurses. J Adv Nurs.

[32] Brewer CS, Kovner CT, Greene W, Tukov-Shuser M, Djukic M (2012). Predictors of actual turnover in a national sample of newly licensed registered nurses employed in hospitals. J Adv Nurs.

[33] Connors CA, Dukhanin V, March AL, Parks JA, Norvell M, Wu AW (2020). Peer support for nurses as second victims: Resilience, burnout, and job satisfaction. J Patient Saf Risk Manag.

[34] Cabilan CJ, Kynoch K (2017). Experiences of and support for nurses as second victims of adverse nursing errors: A qualitative systematic review. JBI Database System Rev Implement Rep.

[35] Tomietto M, Paro E, Sartori R, Maricchio R, Clarizia L, De Lucia P (2019). Work engagement and perceived work ability: An evidence-based model to enhance nurses’ well-being. J Adv Nurs.

[36] Thomas LT, Ganster DC (1995). Impact of family-supportive work variables on work-family conflict and strain: A control perspective. J Appl Psychol.

[37] Gaffney T. Retaining nurses to mitigate shortages: Flexibility, creative policies, and new care models are key to retention. Am Nurse;J2022:14–8.

[38] Daniels RG, McCorkle RJAJ (2016). Design of an evidence-based “second victim” curriculum for nurse anesthetists. AANA J.

[39] Edrees H, Brock DM, Wu AW, McCotter PI, Hofeldt R, Shannon SE (2016). The experiences of risk managers in providing emotional support for healthcare workers after adverse events. J Healthc Risk Manag.

